# Acute ST Elevation Myocardial Infarction in Patients Hospitalized for Non‐Cardiac Conditions: The Next Challenge in Reperfusion Time

**DOI:** 10.1161/JAHA.113.000182

**Published:** 2013-04-24

**Authors:** Thomas M. Todoran, Eric R. Powers

**Affiliations:** 1 Division of Cardiology, Medical University of South Carolina, Charleston, SC (T.M.T., E.R.P.)

**Keywords:** Editorials, reperfusion, ST elevation myocardial infarction, STEMI systems of care

## Introduction

Early animal studies demonstrated that temporary coronary artery occlusion does not lead to irreversible myocardial damage. Delayed reperfusion resulted in a wave of myocardial necrosis extending from the subendocardium to the subepicardium. With increasing occlusion times the amount of transmural necrosis increased.^1^ Further observational studies in humans have demonstrated that early reperfusion therapy can preserve myocardial viability and reduce mortality in patients with acute myocardial infarction. Since the 1980s numerous large randomized trials involving tens of thousands of patients have demonstrated that fibrinolytic therapy can reduce mortality and preserved left ventricular function in patients with suspected acute myocardial infarction.^2^ Furthermore, it has been well established that the earlier reperfusion therapy is started after symptom onset, the larger the mortality reduction, with the absolute mortality reduction greatest among patients who presented within 1 hour of symptom onset.^3^

The benefit of early reperfusion with percutaneous coronary intervention (PCI) has demonstrated incremental improvement in mortality.^4^ The mortality benefit after reperfusion with PCI in patients with acute ST elevation myocardial infarction (STEMI) is the greatest in the first 1 to 3 hours after the onset of symptoms. This is believed to be related to the amount of salvaged myocardium during this time.^5^ With the advent of this therapy, the focus of early reperfusion turned to door‐to‐balloon times. Review of the National Registry of Myocardial Infarction from 1990 to 2006, showed an overall reduction in door‐to‐balloon and door‐to‐needle time for administration of fibrinolytic therapy. There was also a decline in in‐hospital mortality in patients who underwent reperfusion therapy; both fibrinolysis and primary PCI.^6^ This mortality benefit is significantly reduced or even lost beyond 1 hour. Nallamouth and Bates^7^ showed that for every 10 minutes of added delay in performing PCI, the mortality benefit of the PCI compared to fibrinolysis decreased by 1% with the reperfusion strategies becoming equivalent at 62 minutes. Shorter time to reperfusion is associated with smaller infarct size and less microvascular obstruction by cardiac MRI resulting in more salvaged myocardium. After 90 minutes this was markedly reduced.^8^ Longer time to reperfusion is also associated with increase in transmural necrosis.^9^

Metrics for treatment of patients with STEMI have focused on reducing the time to reperfusion after the patient arrives at the hospital. Practice guidelines recommend that time from arrival at the hospital to mechanical reperfusion or “door‐to‐device time” (D2D) be less than 90 minutes.^10^ Potential delays include time to obtain the electrocardiogram (ECG), time to diagnosis of STEMI, time to transport the patient to the cardiac catheterization laboratory, and time for performing the PCI. For patients arriving by ambulance, obtaining a pre‐hospital ECG and allowing emergency department physicians to activate the cardiac catheterization laboratory have been key in streamlining the process and ultimately reducing time to reperfusion.^11^ After implementation of such strategies, there have been marked improvements in median door‐to‐ door to balloon times balloon time nationally. Krumkolz et al,^12^ reported a reduction in D2D from 96 minutes in 2005 to 64 minutes in 2010 in patients with STEMI presenting to primary PCI centers. There were corresponding increases in the percentage of patients who had D2D time <90 minutes (44.2% to 91.4%). In our hospital, the median D2D is now less than 45 minutes with 100% of patients having D2D times of less than 90 minutes.

Recognizing that D2D does not accurately measure coronary occlusion to perfusion time, the key time for myocardial salvage, efforts have been made to further streamline the process by implementing reperfusion strategies that incorporate first medical contact with emergency medical services and transfer protocols to primary PCI centers. A survey from the American Heart Association Mission: Lifeline reports 381 STEMI systems of care in the United States. These systems involve collaboration between emergency medical services (EMS), non‐PCI hospitals, and PCI hospitals.^13^ Implementation of regional STEMI systems of care resulting in further reductions in D2D time can be achieved by increasing pre‐hospital ECGs and direct activation of the cardiac catheterization laboratory by EMS. Expansion of such a system to an entire state has shown increases in pre‐hospital ECGs, modest reduction in median D2D times, and a decline in first medical contact to device time.^14^ This reduction in D2D time observed within regional STEMI systems of care is similar to national trends.^15^

While D2D times have continued to improve nationally with implementations of STEMI systems and improvement in processes of care, it is likely that the benefit of further improvements in D2D will plateau. A recent report from National Cardiovascular Data Registry showed that short‐term mortality is unchanged.^16^ Thus, the new metric for STEMI care has become the first medical contact to reperfusion time. In the future the metric might be symptom onset to reperfusion, as shorter symptom onset to reperfusion times would result in more salvaged myocardium. In a large observational study from Japan, patients presenting within 3 hours of symptom onset had a lower incidence of composite death and congestive heart failure than those presenting later.^17^

One challenge that remains for out‐of‐hospital STEMI is the patient who presents to a non‐PCI hospital either directly or by EMS. Direct transport to a PCI center after prehospital diagnosis of STEMI has been shown to result in a higher percentage of patients having first medical contact to device time <90 minutes than those requiring interfacility transfer.^18^ In well‐developed STEMI systems, first door‐to‐device times within 90 to 120 minutes can be attained for hospitals within 30 minutes transfer‐drive time to a PCI hospital.^19^ For patients outside this window it is possible that a facilitated PCI approach involving initiation of fibrinolysis and transfer to PCI center would be the reperfusion strategy of choice. A recent study demonstrated the value of pre‐hospital fibrinolysis with timely coronary angiography and PCI for patients unable to undergo PCI within 1 hour of first medical contact and presenting within 3 hours of symptom.^20^

In this issue of *JAHA*, Dai et al,^21^ report a single center, retrospective analysis of patients with inpatient STEMI. This study highlights another challenge in STEMI care, the patient hospitalized for noncardiac conditions. The authors identify 2 sources of delay for reperfusion for the patient with inpatient STEMI: recognition of STEMI and time to reperfusion once STEMI was diagnosed. For inpatient STEMI, they report longer median time to obtain ECG (41 versus 5 minutes), ECG to angiography time (91 versus 35 minutes), and ECG to device or reperfusion (140 versus 60 minutes) compared to outpatient STEMI.

Despite these delays, the inpatient STEMI group had lower biomarkers and no difference in ejection fraction compared to the outpatient group. This may be due to a delay in all patients resulting in the time of occlusion to reperfusion being outside the window for significant salvage. The inpatient STEMI group had higher in‐hospital adjusted mortality, 39.6% compared to 4% in the outpatient group. This higher mortality for inpatient STEMI is attributed by the authors to atypical presentation and more comorbid disease. Emergent coronary angiography was performed on 71% of patients with inpatient STEMI of which only 56% underwent PCI. Inpatients who had an ECG within 1 hour were more likely to have symptoms and had a higher rate of revascularization (87% versus 56%). This further illustrates the complexity of these patients. Improvement in the care of the inpatient STEMI might include earlier recognition, earlier ECG acquisition and interpretation along with improved processes to reduce ECG to reperfusion times. However, whether these changes will improve outcomes is unknown. The lack of association between time to reperfusion and myocardial salvage in this study suggests the hypothesis that poor outcomes in inpatients with STEMI are primarily driven by factors other than time to reperfusion.

The present study is an important contribution to insight concerning this next frontier in STEMI care. Further studies addressing this topic are eagerly awaited.
